# Deformed wing virus is not related to honey bees' aggressiveness

**DOI:** 10.1186/1743-422X-3-61

**Published:** 2006-08-30

**Authors:** Agnès Rortais, Diana Tentcheva, Alexandros Papachristoforou, Laurent Gauthier, Gérard Arnold, Marc Edouard Colin, Max Bergoin

**Affiliations:** 1 Laboratoire Populations, Génétique, Evolution CNRS, UPR 9034, avenue de la Terrasse, 91198 Gif-sur-Yvette –, France; 2 Laboratoire de Pathologie Comparée des Invertébrés EPHE, UMR 1231, Biologie Intégrative et Virologie des Insectes INRA, Université Montpellier II, Place Bataillon, 34095 Montpellier –, France; 3 Laboratory of Apiculture-Sericulture, School of Agriculture, Aristotle University of Thessaloniki –, Greece

## Abstract

Guards of Cyprian honey bee colonies, *Apis mellifera cypria*, display a great defensive behaviour against hornets' attacks. The deformed wing virus (DWV) and the kakugo virus (KV) genomes are very similar, but unlike KV, the presence of DWV is not related to honey bees' aggressiveness. This discrepancy is further discussed.

## Findings

Temporal polyethism is widespread among social hymenopteran, and particularly in the honey bee *Apis mellifera *L. [1,2]. The behavioural shifts that occur as a worker ages are associated with physiological changes such as variation in juvenile hormone titres in the insect haemolymph [3] or variation in octopamine levels in the bee head [4]. As the expression patterns of the mRNA in honey bee brains predict behavioural changes [5], one can expect that viral infections located in heads might have profound effects on the behaviour of bees. Until now, only one insect virus – namely the sacbrood virus (SBV), has been found to modify workers tasks. SBV infected adults were found to forage earlier in life than controls, and most infected foragers failed in collecting pollen; these effects were attributed to physiological changes due to viral infection [6,7]. Recently, the kakugo virus (KV), which was only detected in the brain of aggressive workers of Italian bees by real-time PCR, was suggested to trigger behavioural changes in honey bees [8].

Among the 13 honey bee viruses described in *Apis mellifera *L. [9], the deformed wing virus (DWV) is one of the most common [10-12]. DWV belongs to the novel family of the Iflaviridae and its genome consists of a single strand positive RNA encompassing a single open reading frame which codes for both structural and non structural polypeptides [13]. DWV is suspected to induce typical injuries on the wings of infected workers, mostly in those heavily infested with the ectoparasite *Varroa destructor *[9]. In honey bee colonies, association of DWV with mite infestations has been largely documented [10,14-16]. DWV was further evidenced in different worker, queen and drone organs by quantitative RT-PCR and *in situ *hybridisation [17,18] indicating that it might have a considerable degree of tissue specificity.

The DWV and KV sequences show a great homology (98%, at the nucleotide level). Considering this, we tested whether DWV, like KV, is related to honey bees' aggressiveness by comparing DWV RNA loads in aggressive (guards) *versus *non aggressive honey bees (emerging bees, nurses and foragers) issued from several colonies of *Apis mellifera cypria*, a race that exhibits a great defensive behaviour against the hornet *Vespa orientalis *[19].

Honey bees (*Apis mellifera cypria*) were collected in an apiary located in the southern part of Cyprus in three sampling times (October and November 2004, and April 2005). For each sampling time, 10 guards and 10 nurses were collected in 5 colonies (total of 300 individuals), and during the last sampling, 5 more colonies and 2 other temporal castes (emerging bees and foragers) were sampled (total of 100 additional individuals). Emerging bees were collected directly in brood cells and nurses on brood combs. Guards were trapped on flight boards using a living hornet as lure, and foragers were collected when returning to the hive with pollen pellets. All samples were stored in ethanol (95%) and DWV RNA loads were determined by quantitative RT-PCR, using a standardised protocol [16]. Each analysis was representative of a pool of 10 individuals and heads were analysed separately from thoraxes and abdomens.

**Figure 1:**
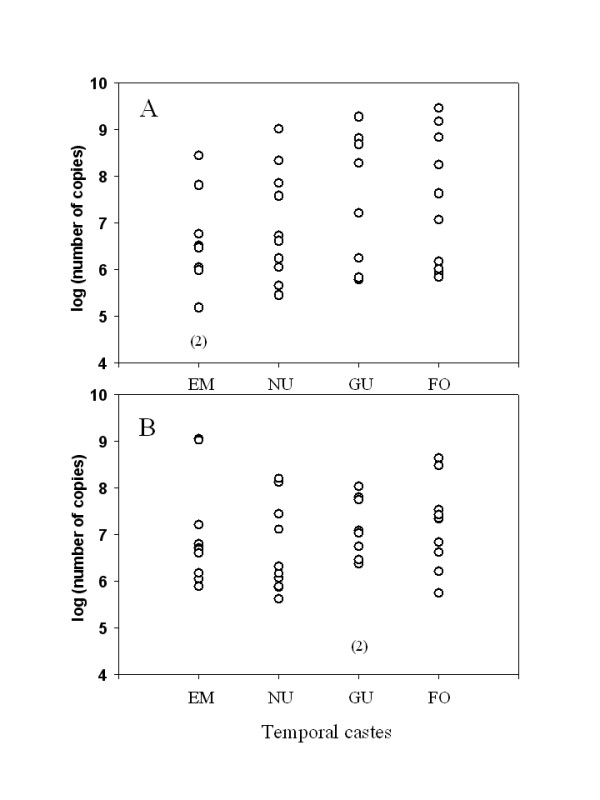
DWV RNA values recorded in the head (A) and body (B) of bees issued from 10 colonies of *A. mellifera cypria *in April 2004. EM: emerging bees; NU: nurses; GU: guards; FO: foragers. Results are representative of 10 individuals and are expressed as mean of triplicates in number of DWV RNA copies per body part (head or thorax and abdomen). The number of negative DWV samples is indicated into brackets.

DWV RNA loads recorded in the different bee samples were compared using the SigmaStat 2.03 software (Systat). A Mann-Whitney Rank Sum Test showed no statistical differences between nurses and guards collected during the three sampling times (in bodies, guards *versus *nurses: T = 294.500, P = 0.428 ; in heads, guards *versus *nurses: T = 350.000, P = 0.820). Likewise, the DWV RNA loads recorded in the 10 colonies collected in April 2004 showed no statistical differences, neither among the four temporal castes nor between body parts (ANOVA on ranks: H = 1.541; df = 3; P = 0.673). Data are presented on Figure 1.

For comparisons, DWV RNA loads were determined in nurses and guards using KV [8] and DWV [16] primers. Each couple of primers displayed similar PCR efficiency (DWV: y = -3.26 x + 17.12; R^2 ^= 0.998 – KV: y = -3.24 x + 17.75; R^2 ^= 0.990), but never allowed to distinguish different viral populations according to the standard error generated by the technique [16].

In this paper, we showed that infection of *A. m. cypria *by DWV is common in all colonies and that there are no significant differences among temporal castes and body parts. Thus, despite their high genome homology, KV and DWV display distinct biological patterns. In order to explain such discrepancies, several hypotheses are proposed.

Small changes in the amino acid composition of DWV and KV might be sufficient to modify their viral tissue tropism such that one variant, the KV, specifically targets the brain of the honey bee and be responsible for the observed behavioural changes. The genome of these two viruses show a higher polymorphism in the putative leader polyprotein coding region which has already been found to be associated with viral pathogenesis [13].

The behaviour of these two viruses may also vary with the origin of honeybees (e.g. race, colony). While KV and DWV are detected in the heads of asymptomatic Italian (*A. m. ligustica*) [8] and Cyprian (*A. m. cypria*) honeybees, respectively, DWV is not detected in the head of asymptomatic German (*A. m. carnica*) honeybees [20]. According to the race and/or the origin of colonies, KV and DWV may not have the same viral tissue tropism. However, given that 10 colonies of *A. m. cypria *honeybees were analysed whereas only two colonies of'*A. m. ligustica *were analysed for the study of KV [8], one cannot exclude that the effects of inter-colonial variations may have been less significant for the detection of KV than for the detection of DWV. This sampling bias poses a serious flaw on the significance of the results previously found [8].

Given the high degree of identity between DWV and KV sequences, it is difficult to design primers specific enough to distinguish between the sequences of the two viruses from a single one bee population. DWV was previously detected by PCR in approximately 60% of colonies [11] suggesting that KV must be absent from 40% of colonies. However, all colonies have guards which aggressivity vary with environmental conditions such as humidity, heat, and nectar availability [21,22]; Thus, it seems very unlikely that the presence of KV or DWV has any relation with the aggressiveness of guards. The absence of any significant relation between the presence of DWV and the aggressiveness of Cyprian honeybees supports this assumption. More likely, the intensity of the defensive reaction depends on interactions between individuals and between environmental and genetic effects (see [23] for a review).

There is now a strong evidence that DWV could spread out among colonies independently of mites infestation, that is by food secretions from nurses to larvae or from queen to workers [18]. The mite, *Varroa destructor*, is responsible for the spreading of DWV in all bee tissues by haemolymph spoliation and reactivation of viral infections [15]. Thus, one can assume that in the absence of the mite, DWV and KV might specifically target bee heads where they further concentrate. Therefore, it is possible that the data recorded by Fujiyuki *et al*. (2004) and those presented in this study reflect different levels of mite infestation among colonies. RNA viruses are generally distributed as quasispecies [24], representing a population made of a cloud of genetic variants. Recently, this population genetic diversity was found to determine pathogenesis through cooperative interactions [25]. It would be challenging to understand why some honey bees colonies (e.g. Cyprian and Italian honey bees) might host particular types of viruses (e.g. DWV and KV) with specific tropisms and different levels of pathogenicity. In that, assuming that DWV replicates in *V. destructor*, one can hypothesizes that mites can influence the genetic outcome of viral populations as it has recently been suggested [20].

## Competing interests

The author(s) declare that they have no competing interests.

## Authors' contributions

AR and DT contributed equally to this work. AR and AP performed field experiments. DT did the quantitative PCR analysis. LG and AR planed the experiments and wrote the manuscript. MEC did the statistical analysis. GA, MEC and MB contributed to the design of the experiments and revised the manuscript. All authors read and approved the final manuscript.
